# Pre-validation Study of the Brazilian Version of the Disruptions in Surgery Index (DiSI) as a Safety Tool in Cardiothoracic Surgery

**DOI:** 10.21470/1678-9741-2017-0141

**Published:** 2017

**Authors:** Vinicius José da Silva Nina, Fabio B. Jatene, Nick Sevdalis, Omar Asdrúbal Vilca Mejía, Carlos Manuel de Almeida Brandão, Rosangela Monteiro, Luiz Fernando Caneo, Paula Gobi Scudeller, Augusto Dimitry Mendes, Vinícius Giuliano Mendes, Bellkiss Wilma Romano

**Affiliations:** 1 Hospital Universitário da Universidade Federal do Maranhão (HUUFMA), São Luís, MA, Brazil.; 2 Instituto do Coração do Hospital das Clínicas da Faculdade de Medicina da Universidade de São Paulo (InCor-HCFMUSP), São Paulo, SP, Brazil.; 3 Institute of Psychiatry, Psychology & Neuroscience, King's College London, London, United Kingdom of Great Britain and Northern Ireland.; 4 Hospital Geral Tarquínio Lopes Filho, São Luís, MA, Brazil.

**Keywords:** Surveys and Questionnaires, Translating, Environment, Medical Errors/Prevention & Control, Patient Care Team/Organization & Administration, Quality Assurance, Health Care, Safety Management

## Abstract

**Introduction:**

Most risk stratification scores used in surgery do not include external and non-technical factors as predictors of morbidity and mortality.

**Objective:**

The present study aimed to translate and adapt transculturally the Brazilian version of the Disruptions in Surgery Index (DiSI) questionnaire, which was developed to capture the self-perception of each member of the surgical team regarding the disruptions that may contribute to error and obstruction of safe surgical flow.

**Methods:**

A universalist approach was adopted to evaluate the conceptual equivalence of items and semantics, which included the following stages: (1) translation of the questionnaire into Portuguese; (2) back translation into English; (3) panel of experts to draft the preliminary version; and (4) pre-test for evaluation of verbal comprehension by the target population of 43 professionals working in cardiothoracic surgery.

**Results:**

The questionnaire was translated into Portuguese and its final version with 29 items obtained 89.6% approval from the panel of experts. The target population evaluated all items as easy to understand. The mean overall clarity and verbal comprehension observed in the pre-test reached 4.48 ± 0.16 out of the maximum value of 5 on the psychometric Likert scale.

**Conclusion:**

Based on the methodology used, the experts' analysis and the results of the pre-test, it is concluded that the essential stages of translation and cross-cultural adaptation of DiSI to the Portuguese language were satisfactorily fulfilled in this study.

**Table:** 

Abbreviations, acronyms & symbols	
DiSI	= Disruptions in Surgery Index
WHO	= World Health Organization

## INTRODUCTION

The last 15 years were characterized by significant changes in clinical and hospital services, especially concerning to safety issues in patient care, which has become the focus of several publications on this topic^[1-5]^.

However, despite numerous interventions to improve patient safety, progress has been slower than originally anticipated. A large-scale study in the USA has shown that error rates have remained relatively constant over the last few years, with one in 10 hospitalized patients likely to suffer an error during hospitalization^[1]^.

In the field of surgical practice, technological and scientific advances have led to a significant increase in the number of procedures worldwide, which are often performed in unsafe conditions, interfering with the promotion and recovery of patients' health^[6]^. The World Health Organization (WHO) estimates that 7,000,000 complications and 1,000,000 deaths occur annually during or immediately after surgery^[7]^.

According to Wachter^[8]^, a high percentage of complications in surgery is due to avoidable adverse events, which are often not related to the lack of technical ability, training or knowledge, but they represent cognitive failures of teamwork. Non-technical skills, such as communication, cooperation, coordination, and leadership, are essential components of teamwork, but limited interpersonal competence is often the underlying cause of adverse events and errors^[9,10]^.

Problems in teamwork that cause interruptions in safe surgical flow are extremely common, with a rate of 17.4 per hour being observed in a cardiac surgery study^[11]^. Such interruptions are defined by Wiegmann et al.^[12]^ as any problem in teamwork, technology/instruments, training, or the environment that results in deviations from the natural progression of an operation, which can potentially compromise the patient safety.

To prevent such errors and minimize interruptions to the flow of working processes in the operating room environment, it is necessary to evaluate the safety culture^[13]^. Most studies that evaluate safety culture in health organizations use questionnaires as a tool for data collection. These questionnaires are based on a combination of dimensions and are considered an efficient strategy because they are anonymous, have low costs and allow to assess professionals' perceptions and behaviors related to safety. They also identify weaknesses and strengths of the safety culture for both staff and hospital, which are indispensable for planning and implementation of improvement interventions^[14,15]^.

Considering that annoyances, disturbances, distractions and interruptions in surgery contribute to error and obstacles to the safe flow of the procedure being performed, the Disruptions in Surgery Index (DiSI) questionnaire was developed to evaluate the professionals' perceptions regarding the environment and its impact on their performance in the operating room^[16]^.

In Brazil, the evaluation of the surgical patient safety culture based on the self-perception of professionals performing the procedures is still incipient; therefore, the cross-cultural translation and adaptation of DiSI questionnaire will bring significant contributions because it will make possible to identify and manage prospectively relevant safety issues in the routines and working conditions of different surgical environments.

Thus, the purpose of this pre-validation study was to analyze the clarity and verbal comprehension of the translated and adapted transculturally Brazilian version of DiSI questionnaire.

## METHODS

The present study was carried out in two phases:

- Phase I - Translation and cross-cultural adaptation
- Phase II - Pre-test of the Brazilian version

### Phase I

#### The Tool

The choice for DiSI was based on three aspects: 1 - free availability obtained by written consent from the author of the original instrument; 2 - perspective of wide use in different cultural contexts; and 3 - psychometric properties of the questionnaire.

DiSI is a tool that has been developed to capture the self-perceptions of operating room staff regarding the disruptions that they and their colleagues have to deal with in the operating room^[16]^.

In DiSI, surgical disruptions are grouped into seven different types: 1 - individual's skill, performance, and personality; 2 - operating room environment; 3 - communication; 4 - coordination and situational awareness; 5 - patient-related disruptions; 6 - team cohesion; and 7 - organizational disruptions. Each disruption type is assessed with two or more specific items.

#### Translation and Adaptation

The translation and adaptation of DiSI were carried out following internationally recommended standards which have been previously adopted in Brazil for cross-cultural adaptation of other health care instruments^[17-20]^.

The semantic evaluation of this instrument was based on the universalist approach which involved four stages: translation, back translation, equivalence appraisal, and criticism by specialists in the thematic area^[17]^.

#### Translation

The instrument was translated from the original, in English, into Portuguese, by two independent Brazilian translators, generating two translations (T1 and T2). Only one of them had knowledge about the phenomenon evaluated by the instrument. Subsequently, the formal evaluation of T1 and T2 equivalence was carried out by five researchers; who, after consensual decisions, prepared a synthesis of the translations (T3). This team was composed of a researcher in Quality of Care and Patient Safety, a nurse with expertise in Health Information, a biologist, and two cardiovascular surgeons.

#### Back Translation

The T3 version was sent for back translation by two independent native English speaker translators, who were unaware of the original instrument, generating two back translations into English (BT1 and BT2), which were compared and approved by a single referee - the author of the original instrument in England.

The evaluations of the translated, back translated and synthesis versions were documented by means of written reports.

#### Appraisal of Equivalence

The equivalence was evaluated by a panel of experts based on the original, T1, T2, T3, BT1, BT2, and the respective reports generated during the translation and synthesis stages.

The decisions about the equivalence between the original source and the target version were made by this panel considering four aspects:

- Semantic Equivalence: to evaluate if the translated words meant the same thing; if there were multiple meanings of a given item and if there were grammatical difficulties in translation;
- Idiomatic Equivalence: sought to identify if equivalent expressions were formulated in the target version, avoiding difficulties in translating colloquialisms and idiomatic expressions;
- Empirical Equivalence: evaluated whether we substituted terms in the questionnaire for similar ones that are used in our culture, seeking to capture experiences of daily life;
- Conceptual Equivalence: allowed to observe if the words had different meanings between the cultures and to replace the inadequate terms.

#### Criticism by Specialists in the Thematic Area

The expert committee consisted of the four researchers involved in the study, a linguist specialist, one of the translators, two specialists in patient safety and quality, and a psychologist with experience in Psychometric Assessment of Questionnaires.

The committee's work consisted in detecting possible divergences in the translations, by comparing the terms and words to each other, and also verifying whether the items of the translated instrument referred or not to the concepts measured in the original instrument. The descriptors accepted by at least 80% of the specialists were considered as having adequate translation. Based on expert opinions, the final version of the instrument (T4) was created.

### Phase II

#### Pre-test

The pre-test was performed to evaluate the verbal comprehension and clarity of the questions using a non-random sample of 43 professionals divided into three subgroups that compose a typical team of cardiovascular and thoracic operating rooms of a University Hospital: 9 surgeons, 26 nurses/technicians, and 8 anesthesiologists.

They were asked to indicate in a protocol sheet how much they understood of each item, using a Likert scale:

0 - I did not understand anything;
1 - I understood only a little;
2 - I understood more or less;
3 - I understood almost everything, but I had some doubts;
4 - I understood almost everything;
5 - I understood perfectly and I had no doubts.

**Fig. 1:**
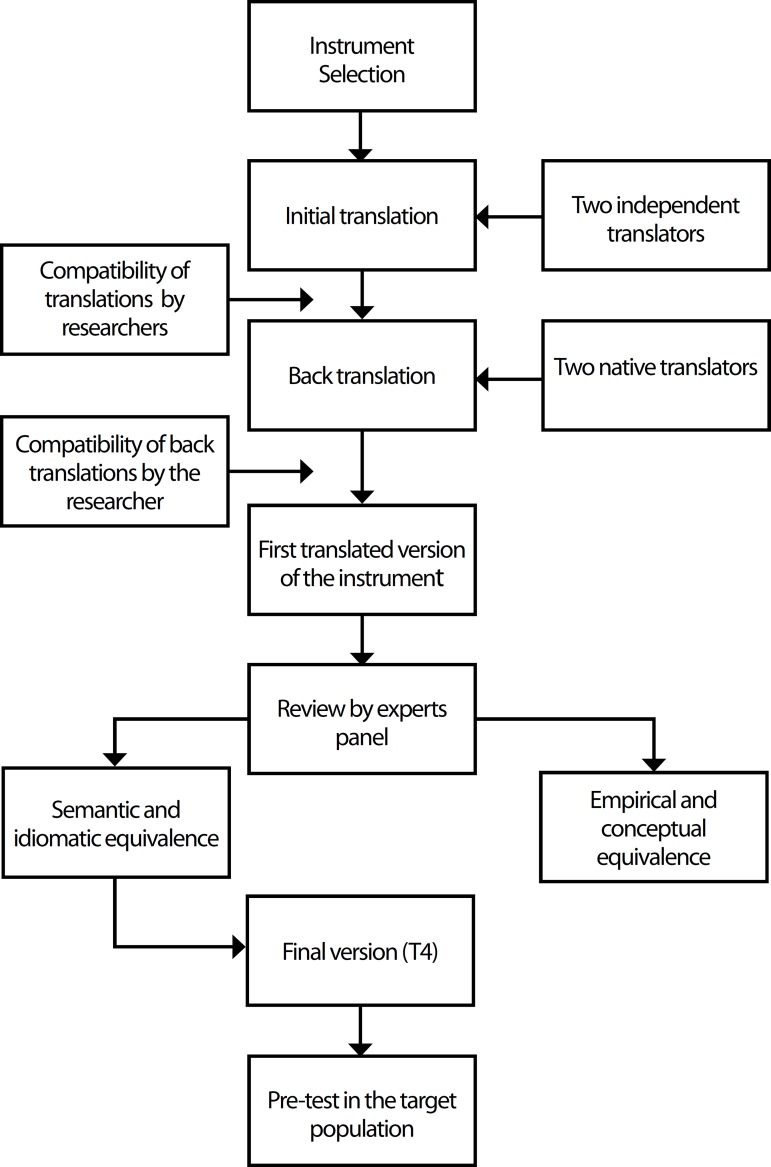
Flowchart showing the steps for translation and crosscultural adaptation of the Disruptions in Surgery Index (DiSI).

Responses 0, 1, 2 and 3 indicated insufficient understanding. If the overall mean of comprehension was ≥ 4.0 (maximum value = 5), it indicated that the questions were easy to understand and that the instrument would be ready to be validated^[15,17]^. The phases of the study are shown in Figure 1.

### Statistical Analysis

Descriptive analyses of the pre-test data were performed using software Stata version 12.0 (Statacorp LP, College Station, Texas, United States). The qualitative variables were expressed by frequencies and percentages. The quantitative variables from the psychometric Likert scale applied to the target population to assess overall clarity and verbal comprehension of the translated version were expressed by means and standard deviations.

### Ethical Aspects

In accordance with the regulatory guidelines for research involving human beings, this study was approved by the Institutional Research Ethics Committee under Consubstantiated Act No. 1,650,066 as of 07/27/2016.

All of the professionals who agreed to participate in the study signed the Informed Consent Form.

## RESULTS

### Translation and Adaptation

**Fig. 2:**
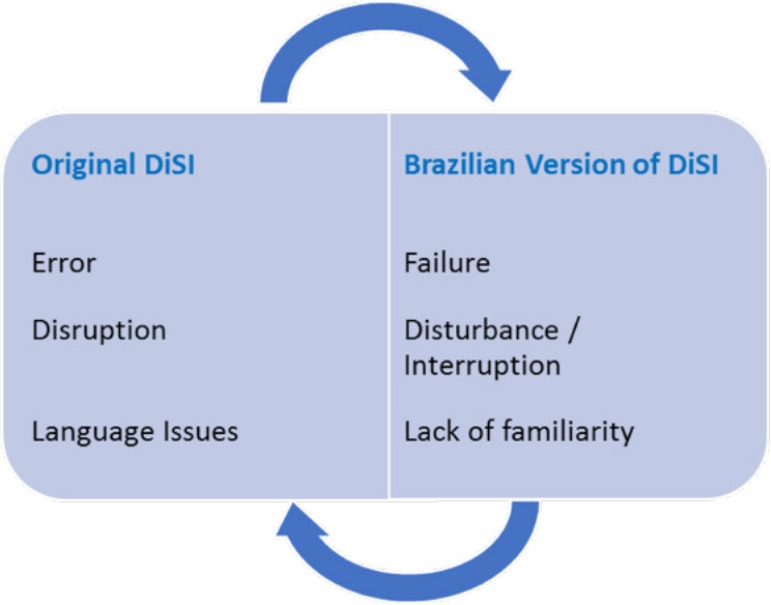
Cross-cultural adaptation of divergent descriptors. DiSI =Disruptions in Surgery Index.

The final Brazilian version of DiSI was approved with 89.6% (23 of 26 descriptors) agreement among the experts and representatives of the target population that composed the panel of experts; however, during the process of cross-cultural adaptation, empirical and conceptual divergences were observed in three (10.4%) of the descriptors (Figure 2).

The empirical and conceptual divergences and the crosscultural adaptations with their respective justifications by the Panel of Experts are described below:

- The word "error" in our culture denotes "guilt"; although the questionnaire is anonymous, this could inhibit respondents from pointing out a true response, so the word "failure" was chosen to replace "error";
- "Disruption" has been replaced by "disturbance/interruption". The committee considered that not all disturbances lead to an interruption in the flow of the operation, but any disruption originates from an initial disturbance;
- The term "language issues" has been adapted as a "lack of familiarity" of some employees with the terminology used in the operating room which can lead to interruptions of the surgery because of linguistic misunderstandings;

These divergences, after being discussed and adapted by the panel of experts, were approved in consensus with the developer of the original instrument in England (Sevdalis et al.^[16]^), who ratified that there was a semantic equivalence between the English source version and the Target version in Portuguese.

For being widely recognized and used in the scientific community, the title of the instrument remained in English in the final version. The panel of experts also considered that there was operational equivalence between the format of the original questionnaire and that of the translated and adapted target instrument which was maintained for the pre-test (Appendix A).

### Pre-test

The pre-test was performed with a target population of 43 professionals (62.8% female), whose main area of practice was cardiothoracic surgery: 9 surgeons, 8 anesthesiologists and 26 nurses/technicians. The mean age was 36.8±9.95 years and the mean practice time was 11.5±9.26 years.

By analyzing the Likert scale responses, it was observed that the 29 questions distributed in the seven domains (A-G) of translated and adapted version of DiSI were easy to understand. The overall mean comprehension reached 4.48 ± 0.16. The mean values of all items of the questionnaire presented verbal comprehension greater than 4.0 (maximum = 5) as described in Table 1.

**Table 1:** Mean verbal comprehension of all items of the Disruptions in Surgery Index (DiSI) after translation and crosscultural adaptation. São Paulo, 2017.

Items	Mean	Standard deviation
A	4.51	0.8
A1	4.69	0.6
A2	4.61	0.79
A3	4.58	0.79
A4	4.55	0.73
A5	4.18	1.23
B	4.62	0.68
B1	4.62	0.65
B2	4.61	0.69
B3	4.67	0.6
B4	4.62	0.69
B5	4.55	0.85
B6	4.74	0.58
C	4.54	0.95
C1	4.46	0.93
C2	4.16	1.11
D	4.55	0.87
D1	4.6	0.76
D2	4,32	1.04
D3	4,65	0.65
D4	4.53	0.73
D5	4.18	1.09
D6	4.41	0.9
E	4.51	0.85
E1	4.53	0.93
E2	4.46	1.07
E3	4.45	0.99
E4	4.51	0.96
F	4.5	0.89
F1	4.48	1
F2	4.33	1.22
G	4.61	0.88
G1	4.43	1.07
G2	4.3	1.02
G3	4.35	0.9
G4	4.07	1.36
Total	4,4855556	0,159721981

A=individuals' skill, performance, and personality; B=operating room environment; C=communication; D=coordination and situational awareness; E=patient-related disruptions; F=team cohesion; G=organizational disruptions

## DISCUSSION

In general, the risk stratification scores adapted to different scenarios allow estimating the patient's operative risk and evaluating the results and, eventually, an institution's quality of care^[21-23]^. However, they do not include external and nontechnical factors as predictors of morbidity and mortality that are based on the self-perception of professionals in the operating room.

The present study aimed to fill this knowledge gap by translating and adapting the DiSI questionnaire to the Brazilian version which was developed to capture the self-perception of each member of the surgical team regarding the disruptions they and their colleagues have to deal with in the operating room by judging how often each disruption contributes to an error or obstruction of safe surgical flow^[16]^.

Transcultural adaptation of a self-administered questionnaire to be used in a new country, culture and/or language requires a specific methodology to achieve equivalence between the original source and the target version in order to maintain the content validity of the instrument on a conceptual level between different cultures. Thus, a robust methodology based on international guidelines and associated with interventions and suggestions of the experts was used in the development of the Brazilian version of DiSI^[15,17,19,20,24-26]^.

The translation and adaptation process carried out in the present study involved some complex topics, such as the conceptual adequacy and adaptation of the words *error* and *disruptions* and the expression l*anguage issues*. These were necessary to guarantee the semantic equivalence and, therefore, the understanding of the expressed content in the original instrument and in the the Brazilian version. Despite these three divergences, the final version was approved by 89.6% of the experts, being superior to that recommended in the international literature (80%)^[17,24,25]^.

It is recommended that in the final stage of the adaptation process a pre-test of a new questionnaire be performed in 30 to 40 individuals of the target population with the objective of evaluating the verbal comprehension and clarity of the instrument^[17]^. Thus, a field study with 43 professionals, whose main area of practice was cardiothoracic surgery, was conducted by the authors using Likert's psychometric scale and reaching an overall mean comprehension of 4.48 ± 0.16. These results are similar to those of other authors who used a similar methodology to translate and adapt questionnaires for application in Brazil^[15,19,20,27]^.

### Limitations

This is a pre-validation study whose specific methodology does not foresee the analysis of the constructs validity and reliability. However, it contributes to the evidence that the adapted version retained its equivalence from both the specialists in that thematic area and from the target population in which it was applied.

## CONCLUSION

Based on the methodology used as recommended by the international guidelines, on the technical discussions with experts and also on the results of the pre-test, it is concluded that the essential stages of DiSI translation and cross-cultural adaptation to the Portuguese language were satisfactorily fulfilled in this study.

**Table:** 

Authors' roles & responsibilities	
VJSN	Conception and study design; manuscript writing; final manuscript approval
FBJ	Conception and study design; final manuscript approval
NS	Critical review of the manuscript; final manuscript approval
OAVM	Study design; analysis and data interpretation; final manuscript approval
CMAB	Study design; analysis and data interpretation; final manuscript approval
RM	Study design; analysis and data interpretation; final manuscript approval
LFC	Study design; analysis and data interpretation; final manuscript approval
PGS	Study design; analysis and data interpretation; final manuscript approval
ADM	Statistical analysis; data acquisition and interpretation; final manuscript approval
VGM	Statistical analysis; data interpretation and final manuscript approval
BW	Study design; analysis and data interpretation; final manuscript approval

## Disruptions in Surgery Index (DiSI)

### INSTRUÇÕES

Esta pesquisa capta percepções individuais de perturbações e/ou interrupções com as quais **VOCÊ** e **SEUS COLEGAS** têm que lidar na sala de cirurgia. Considerando-se que uma perturbação e/ou interrupção contribui para a ocorrência de erros e/ou impede que os objetivos do procedimento sejam atingidos, solicitamos que dê as pontuações duas vezes: uma vez por você e mais uma vez por seus colegas. Observe que a perspectiva dos seus colegas se refere a toda a equipe da sala de cirurgia e não apenas dos seus pares.

- Quando perguntamos com que frequência, em média, você e seus colegas observam uma determinada perturbação e/ou interrupção na Sala de Cirurgia, solicitamos que a resposta seja dada utilizando a
- Quando perguntamos o quanto cada perturbação e/ou interrupção contribui potencialmente para que você ou seus colegas cometam um erro ou até que ponto cada perturbação e/ou interrupção impede que os objetivos do procedimento sejam atingidos na sua perspectiva e na perspectiva dos seus colegas, solicitamos que a resposta seja dada utilizando a

Pense em um dia comum na Sala de Cirurgia deste hospital no mês passado.

Com que frequência, em média, você acha que as situações abaixo ocorrem:

**Table:**

Tipo de perturbação e/ou interrupção	Pense em VOCÊ																					Pense em TODOS OS SEUS COLEGAS DO CENTRO CIRÚRGICO																				
**A. Habilidade individual, desempenho e personalidade**	**% de vezes que isso ocorre com você**	**O quanto isso contribui para que você cometa uma falha na Sala de Cirurgia?**										**O quanto isso impede que as metas do procedimento sejam atingidas sob o seu ponto de vista?**										**% de vezes que isso ocorre com eles**	**O quanto isso contribui para que eles cometam uma falha na Sala de Cirurgia?**										**O quanto isso impede que as metas do procedimento sejam atingidas sob o ponto de vista deles?**									
**A1. Estar cansado**		0	1	2	3	4	5	6	7	8	9	0	1	2	3	4	5	6	7	8	9		0	1	2	3	4	5	6	7	8	9	0	1	2	3	4	5	6	7	8	9
**A2. *Ter falhas de atenção***		0	1	2	3	4	5	6	7	8	9	0	1	2	3	4	5	6	7	8	9		0	1	2	3	4	5	6	7	8	9	0	1	2	3	4	5	6	7	8	9
**A3. *Perdera paciência com facilidade***		0	1	2	3	4	5	6	7	8	9	0	1	2	3	4	5	6	7	8	9		0	1	2	3	4	5	6	7	8	9	0	1	2	3	4	5	6	7	8	9
**A4. *Ter excesso de confiança***		0	1	2	3	4	5	6	7	8	9	0	1	2	3	4	5	6	7	8	9		0	1	2	3	4	5	6	7	8	9	0	1	2	3	4	5	6	7	8	9
**A5. *Sentir falta de uma avaliação sobre o desempenho***		0	1	2	3	4	5	6	7	8	9	0	1	2	3	4	5	6	7	8	9		0	1	2	3	4	5	6	7	8	9	0	1	2	3	4	5	6	7	8	9
**B. Ambiente da Sala de Cirurgia**	**% de vezes que isso ocorre com você**	**O quanto isso contribui para que você cometa falha na Sala de Cirurgia?**										**O quanto isso impede que as metas do procedimento sejam atingidas sob o seu ponto de vista?**										**% de vezes que isso ocorre com eles**	**O quanto isso contribui para que eles cometam falha na Sala de Cirurgia?**										**O quanto isso impede que as metas do procedimento sejam atingidas sob o ponto de vista deles?**									
**B1. *Ficar irritado com bipes estridentes e repetitivos***		0	1	2	3	4	5	6	7	8	9	0	1	2	3	4	5	6	7	8	9		0	1	2	3	4	5	6	7	8	9	0	1	2	3	4	5	6	7	8	9
**B2. *Ser distraído por ruido externo***		0	1	2	3	4	5	6	7	8	9	0	1	2	3	4	5	6	7	8	9		0	1	2	3	4	5	6	7	8	9	0	1	2	3	4	5	6	7	8	9
**B3. Ser distraído por música alta**		0	1	2	3	4	5	6	7	8	9	0	1	2	3	4	5	6	7	8	9		0	1	2	3	4	5	6	7	8	9	0	1	2	3	4	5	6	7	8	9
**B4. *Serdistraido por pessoas entrando e saindo da sala de cirurgia***		0	1	2	3	4	5	6	7	8	9	0	1	2	3	4	5	6	7	8	9		0	1	2	3	4	5	6	7	8	9	0	1	2	3	4	5	6	7	8	9
**B5. *Ficar irritado com a temperatura***		0	1	2	3	4	5	6	7	8	9	0	1	2	3	4	5	6	7	8	9		0	1	2	3	4	5	6	7	8	9	0	1	2	3	4	5	6	7	8	9
**B6. *Ficar irritado pela falta ou não funcionamento de equipamento***		0	1	2	3	4	5	6	7	8	9	0	1	2	3	4	5	6	7	8	9		0	1	2	3	4	5	6	7	8	9	0	1	2	3	4	5	6	7	8	9
**C. Comunicação**	**% de vezes que isso ocorre com você**	**O quanto isso contribui para que você cometa uma falha na Sala de Cirurgia?**										**O quanto isso impede que as metas do procedimento sejam atingidas sob o seu ponto de vista?**										**% de vezes que isso ocorre com eles**	**O quanto isso contribui para que eles cometam falha na Sala de Cirurgia?**										**O quanto isso impede que as metas do procedimento sejam atingidas sob o ponto de vista deles?**									
**C1. *Ser distraído por bate-papo irrelevante***		0	1	2	3	4	5	6	7	8	9	0	1	2	3	4	5	6	7	8	9		0	1	2	3	4	5	6	7	8	9	0	1	2	3	4	5	6	7	8	9
**C2. *Ser incomodado por dificuldade com a compreensão da linguagem***		0	1	2	3	4	5	6	7	8	9	0	1	2	3	4	5	6	7	8	9		0	1	2	3	4	5	6	7	8	9	0	1	2	3	4	5	6	7	8	9
**D. Coordenação e percepção da situação**	**% de vezes que isso ocorre com você**	**O quanto isso contribui para que você cometa uma falha na Sala de Cirurgia?**										**O quanto isso impede que as metas do procedimento sejam atingidas sob o seu ponto de vista?**										**% de vezes que isso ocorre com eles**	**O quanto isso contribui para que eles cometam uma falha na Sala de Cirurgia?**										**O quanto isso impede que as metas do procedimento sejam atingidas sob o ponto de vista deles?**									
**D1. *Ficar irritado com alterações de última hora na programação cirúrqica***		0	1	2	3	4	5	6	7	8	9	0	1	2	3	4	5	6	7	8	9		0	1	2	3	4	5	6	7	8	9	0	1	2	3	4	5	6	7	8	9
**D2. *Ficar irritado com a preparação da(s) próxima(s) cirurqia(s)***		0	1	2	3	4	5	6	7	8	9	0	1	2	3	4	5	6	7	8	9		0	1	2	3	4	5	6	7	8	9	0	1	2	3	4	5	6	7	8	9
**D3.* Ficar irritado com atrasos de membros da equipe***		0	1	2	3	4	5	6	7	8	9	0	1	2	3	4	5	6	7	8	9		0	1	2	3	4	5	6	7	8	9	0	1	2	3	4	5	6	7	8	9
**D4. *Ficar irritado com a ausência de membros da equipe durante o procedimento***		0	1	2	3	4	5	6	7	8	9	0	1	2	3	4	5	6	7	8	9		0	1	2	3	4	5	6	7	8	9	0	1	2	3	4	5	6	7	8	9
**D5. *Ser distraído pela falta de conhecimento da dinâmica do trabalho em equipe***		0	1	2	3	4	5	6	7	8	9	0	1	2	3	4	5	6	7	8	9		0	1	2	3	4	5	6	7	8	9	0	1	2	3	4	5	6	7	8	9
**D6. *Ser distraído pela realização de várias tarefas ao mesmo tempo***		0	1	2	3	4	5	6	7	8	9	0	1	2	3	4	5	6	7	8	9		0	1	2	3	4	5	6	7	8	9	0	1	2	3	4	5	6	7	8	9
**E. Perturbações e/ou interrupções relacionadas ao paciente**	**% de vezes que isso ocorre com vocè**	**O quanto isso contribui para que você cometa falha na Sala de Cirurgia?**										**O quanto isso impede que as metas do procedimento sejam atingidas sob o seu ponto de vista?**										**% de vezes que isso ocorre com eles**	**O quanto isso contribui para que eles cometam uma falha na Sala de Cirurgia?**										**O quanto isso impede que as metas do procedimento sejam atingidas sob o ponto de vista deles?**									
**E1. *Ficar irritado pela falta de informações necessárias sobre o paciente***		0	1	2	3	4	5	6	7	8	9	0	1	2	3	4	5	6	7	8	9		0	1	2	3	4	5	6	7	8	9	0	1	2	3	4	5	6	7	8	9
**E2. *Ficar irritado com informações imprecisas sobre o paciente***		0	1	2	3	4	5	6	7	8	9	0	1	2	3	4	5	6	7	8	9		0	1	2	3	4	5	6	7	8	9	0	1	2	3	4	5	6	7	8	9
**E3. *Ficar irritado com a indisponibilidade de registro de informações preoperatorias***		0	1	2	3	4	5	6	7	8	9	0	1	2	3	4	5	6	7	8	9		0	1	2	3	4	5	6	7	8	9	0	1	2	3	4	5	6	7	8	9
**E4. *Ficar irritado com a indisponibilidade de resultados de exames***		0	1	2	3	4	5	6	7	8	9	0	1	2	3	4	5	6	7	8	9		0	1	2	3	4	5	6	7	8	9	0	1	2	3	4	5	6	7	8	9
**F. Coesão da equipe**	**% de vezes que isso ocorre com você**	**O quanto isso contribui para que você cometa uma falha na Sala de Cirurgia?**										**O quanto isso impede que as metas do procedimento sejam atingidas sob o seu ponto de vista?**										**% de vezes que isso ocorre com eles**	**O quanto isso contribui para que eles cometam uma falha na Sala de Cirurgia?**										**O quanto isso impede que as metas do procedimento sejam atingidas sob o ponto de vista deles?**									
**F1.* Não se sentir como parte da equipe***		0	1	2	3	4	5	6	7	8	9	0	1	2	3	4	5	6	7	8	9		0	1	2	3	4	5	6	7	8	9	0	1	2	3	4	5	6	7	8	9
**F2. *Sentir-se com o moral baixo***		0	1	2	3	4	5	6	7	8	9	0	1	2	3	4	5	6	7	8	9		0	1	2	3	4	5	6	7	8	9	0	1	2	3	4	5	6	7	8	9
**G. Perturbações e/ou interrupções organizacionais**	**% de vezes que isso ocorre com você**	**O quanto isso contribui para que você cometa uma falha na Sala de Cirurgia?**										**O quanto isso impede que as metas do procedimento sejam atingidas sob o seu ponto de vista?**										**% de vezes que isso ocorre com eles**	**O quanto isso contribui para que eles cometam uma falha na Sala de Cirurgia?**										**O quanto isso impede que as metas do procedimento sejam atingidas sob o ponto de vista deles?**									
**G1. *Ensinar causa distração***		0	1	2	3	4	5	6	7	8	9	0	1	2	3	4	5	6	7	8	9		0	1	2	3	4	5	6	7	8	9	0	1	2	3	4	5	6	7	8	9
**G2. *Pressão de tempo causa distração***		0	1	2	3	4	5	6	7	8	9	0	1	2	3	4	5	6	7	8	9		0	1	2	3	4	5	6	7	8	9	0	1	2	3	4	5	6	7	8	9
**G3. *Ficar irritado com as políticas de racionamento do hospital***		0	1	2	3	4	5	6	7	8	9	0	1	2	3	4	5	6	7	8	9		0	1	2	3	4	5	6	7	8	9	0	1	2	3	4	5	6	7	8	9
**G4. *Ficar irritado com programação de cirurgia não realista***		0	1	2	3	4	5	6	7	8	9	0	1	2	3	4	5	6	7	8	9		0	1	2	3	4	5	6	7	8	9	0	1	2	3	4	5	6	7	8	9
